# β-Blocker after severe traumatic brain injury is associated with better long-term functional outcome: a matched case control study

**DOI:** 10.1007/s00068-017-0779-5

**Published:** 2017-03-08

**Authors:** R. Ahl, E. P. Thelin, G. Sjölin, B.-M. Bellander, L. Riddez, P. Talving, S. Mohseni

**Affiliations:** 1 Division of Trauma and Emergency Surgery, Department of Surgery, Karolinska University Hospital, 171 76 Stockholm, Sweden; 2 Division of Trauma and Emergency Surgery, Department of Surgery, Orebro University Hospital, 701 85 Orebro, Sweden; 3 School of Medical Sciences, Orebro University, Orebro, Sweden; 4 Department of Clinical Neuroscience, Karolinska Institutet, Solna, 17176 Stockholm, Sweden; 5 Department of Surgery, Tartu University Hospital, Puusepa 8, Tartu, 50406 Estonia

**Keywords:** Beta-blocker, Traumatic brain injury, Functional outcome

## Abstract

**Purpose:**

Severe traumatic brain injury (TBI) is the predominant cause of death and disability following trauma. Several studies have observed improved survival in TBI patients exposed to β-blockers, however, the effect on functional outcome is poorly documented.

**Methods:**

Adult patients with severe TBI (head AIS ≥ 3) were identified from a prospectively collected TBI database over a 5-year period. Patients with neurosurgical ICU length of stay <48 h and those dying within 48 h of admission were excluded. Patients exposed to β-blockers ≤ 48 h after admission and who continued with treatment until discharge constituted β-blocked cases and were matched to non β-blocked controls using propensity score matching. The outcome of interest was Glasgow Outcome Scores (GOS), as a measure of functional outcome up to 12 months after injury. GOS ≤ 3 was considered a poor outcome. Bivariate analysis was deployed to determine differences between groups. Odds ratio and 95% CI were used to assess the effect of β-blockers on GOS.

**Results:**

362 patients met the inclusion criteria with 21% receiving β-blockers during admission. After propensity matching, 76 matched pairs were available for analysis. There were no statistical differences in any variables included in the analysis. Mean hospital length of stay was shorter in the β-blocked cases (18.0 vs. 26.8 days, *p* < 0.01). The risk of poor long-term functional outcome was more than doubled in non-β-blocked controls (OR 2.44, 95% CI 1.01–6.03, *p* = 0.03).

**Conclusion:**

Exposure to β-blockers in patients with severe TBI appears to improve functional outcome. Further prospective randomized trials are warranted.

## Background

Severe traumatic brain injury (TBI) is the predominant cause of death and disability following trauma. The incidence of TBI is increasing, a trend which is particularly pertinent amongst elderly [1]. Patients who survive TBI often experience long-term neurological impairment. Severe functional disability following TBI is a global public health concern [2].

Multiple risk factors have been associated with an increased morbidity and mortality following TBI including increasing age, diminished Glasgow Coma Scale (GCS) score on admission, episodes of hypotension or hypoxia and increasing Injury Severity Score (ISS) [2–5]. Early interventions after TBI are of paramount importance, nevertheless, despite extensive research, there are few evidence-based interventions for patients with severe brain injury resulting in improved long-term functional outcomes [6, 7].

Previous studies have noted associations between non-neurological complications and the catecholamine surge that occur at the time of cerebral insult, which appears proportional to the degree of brain injury [8–10]. It has been postulated that β-blockers might down-regulate the potential toxic effects of the ‘sympathetic storm’ following TBI. Sympathetic hyperactivity is thought to increase cerebral vasoconstriction, which is believed to contribute to local edema and increased intracranial pressure. These detrimental events facilitate the progression of secondary brain injury with reduced brain perfusion and oxygenation resulting in increased overall mortality and disability [11]. Consequently, abating the trauma-induced sympathetic storm has the potential to reduce secondary insults and thereby improve immediate and long-term functional outcome. Several clinical studies, along with some prospective experimental investigations, have demonstrated promising effects of β-blockade on the overall outcome following TBI [12–17]. These studies have, however, focused mainly on in-hospital mortality rates. We set out to study the effects of β-blockade in patients experiencing severe TBI with the hypothesis that β-blocker therapy may improve functional outcome in these instances.

## Materials and methods

The current study has been approved by the ethics committee and has been performed in accordance with the ethical standards laid down in the Declaration of Helsinki. After IRB approval, the traumatic brain injury database of the Neurosurgery Department at Karolinska University Hospital, Stockholm, Sweden, was queried for adult patients (age ≥18 years) sustaining serious to critical TBI (head AIS ≥ 3) admitted between 1/2007 and 12/2011. Given the aim of evaluating the impact of early β-blockade on long-term functional outcome, patients were stratified to the β-blocked cases when exposed to β-blockers within 48 h of admission and were continued on β-blockers until discharge from hospital. Patients not receiving β-blockers constituted the control cohort in this study. Patients suffering penetrating cerebral trauma were excluded. Patients with a neurosurgical intensive care unit (NICU) length of stay (LOS) of less than 48 h and those with a head AIS of six or those not surviving beyond 2 days following admission were excluded from the study to control for early deaths due to non-survivable injuries.

Patient data were obtained from the TBI registry containing prospectively collected data, including age, gender, admission GCS score, admission systolic blood pressure (SBP), imaging results including computed tomography (CT) or magnetic resonance imaging (MRI), head Abbreviated Injury Scale (AIS), New Injury Severity Score (NISS), Charlson’s Comorbidity Index, β-blocker exposure, the time and type of β-blocker administered, neurosurgical interventions, NICU LOS, hospital LOS, and mortality. All cases with pertinent ICD-10 codes were reviewed to ensure that the AIS ≥ 3 was attributed to an intracranial injury (ICD-10 codes S06.1–S06.9). Admission CT scan was defined according to Rotterdam CT classification scores [18]. Glasgow Outcome Score (GOS) at discharge and up to 12 months following trauma was acquired to assess functional outcome (Table 1). At the study site, GOS is regularly assessed at discharge and at 3–6 months post injury at a follow-up appointment at the neurosurgical or rehabilitation clinic. Finally, 12 months after injury, a questionnaire regarding quality of life (TBI-QOL) was sent out to patients. In the current study, GOS at 12 months after injury was utilized to document long-term functional outcome. A GOS of ≤ 3 was considered to be a poor outcome. The patients included in the study were managed per guidelines set forth by the Brain Trauma Foundation [19].

**Table 1:** Classification of the Glasgow Outcome Score (GOS)

Glasgow Outcome Score	Definition
GOS 1	Dead
GOS 2	Vegetative state
GOS 3	Severe disability, dependent state
GOS 4	Moderate disability, independent state
GOS 5	Mild disability, full recovery

### Statistical analysis

The primary outcome of interest was GOS at discharge and up to 12 months following discharge. For analysis purposes, several continuous variables were dichotomized using clinically relevant cut-off points (age ≥ 55 vs. <55 years, SBP ≥ 90 vs. <90 mmHg, GCS >8 vs. ≤ 8, head AIS = 3 vs. ≥ 4, and GOS ≤ 3 vs. ≥ 4). Since the number of confounders was large in comparison with the number of events, cases receiving β-blocker therapy were matched in a 1:1 ratio to control cases that were not receiving such therapy using propensity score matching. Variables included in the propensity score model were age, gender, NISS, GCS, intracranial injury characteristics, head AIS, Rotterdam CT score, and Charlson’s Comorbidity Index. Propensity scores (predicted probability of receiving β-blockers) were calculated for all patients using binary logistic regression [20]. Each patient receiving β-blockers was matched to a control not subjected to β-blockers within a 0.0135 caliper of propensity without replacement. The caliper was equal to one-quarter of a standard deviation (SD) of the logit of the propensity score [21].

Demographic information and clinical characteristics between the matched cohorts were compared using bivariate analysis. The differences between the cohorts were tested for significance using McNemar’s test for categorical variables and paired Student’s *t* test or Wilcoxon signed rank test for continuous variables when appropriate. Values are reported as percentages for categorical variables and mean (SD) or median (quartiles) for continuous variables. The difference in risk of suffering an unfavorable GOS (≤ 3) between groups was analyzed and the odds ratio (OR) and 95% confidence interval (CI) were derived. Values were considered statistically significant at a two-tailed *p* value of <0.05. The statistical analysis was performed using the Statistical Package for Social Science (SPSS Windows©) version 21.0 (SPPS Inc., Chicago, IL).

## Results

During the 5-year study period, a total of 362 patients met the inclusion criteria. Of these, 76 (21.0%) patients received β-blockers during their hospital admission. After propensity matching, 76 matched pairs (*n* = 152) were available for analysis. The average age of patients was 58 ± 16 years and 77.0% were male. At admission, a total of 48.7% of patients had a GCS of ≤ 8 and 84.9% had a head AIS ≥ 4 (Table 2). When excluding head AIS scores from the calculation of NISS (non-head NISS) to estimate the burden of extracranial injuries in the cohort, 89.5% of patients had a non-head NISS ≤ 9 and only 6.6% of patients had a non-head NISS exceeding fifteen. Subdural hemorrhage was the most common type of intracranial injury (65.1%). Intracranial pressure monitoring was used in 63.8% of the study cohort and 54.6% underwent evacuation surgery (Table 2).

**Table 2:** Demographic, clinical information, and outcomes for the total cohort

Variable	Total cohort (*n* = 152)
Patient characteristics	
Male gender	77.0% (117)
Age in years	
Mean (SD)	57.5 (16.0)
Median (LQ, UQ)	62 (49, 69)
Age ≥ 55 years	70.4% (107)
CCI	
Mean (SD)	3.4 (2.6)
Median (LQ, UQ)	3 (2, 5)
Specific intracranial injury	
Diffuse axonal injury	5.3% (8)
Focal injury	11.8% (18)
Epidural hemorrhage	11.8% (18)
Subdural hemorrhage	65.1% (99)
Subarachnoid hemorrhage	5.3% (8)
Injury severity	
Hypotension (<90 mmHg)	3.3% (5)
GCS ≤ 8	48.7% (74)
Head AIS ≥ 4	84.9% (129)
Non-head NISS ≤ 9	89.5 (136)
Rotterdam, median (LQ, UQ)	4 (3, 5)
Neurosurgical interventions	
ICP monitoring	63.8% (97)
Operation	54.6% (83)
Outcomes	
NICU LOS	
Mean (SD), days	9.6 (8.8)
Median (LQ, UQ), days	7 (3, 15)
Hospital LOS	
Mean (SD), days	22.4 (18.3)
Median (LQ, UQ), days	17 (8, 30)
In-hospital mortality	9.9% (15)
12-month mortality	24.3% (37)
GOS ≤ 3 at discharge	88.8% (135)
GOS ≤ 3 at follow-up	50.7% (77)

*GCS* Glasgow Coma Scale, *CCI* Charlson’s Comorbidity Index, *AIS* abbreviated injury scale, *NISS* New Injury Severity Score, *ICP* intracranial pressure, *NICU* neurosurgical intensive care unit, *LOS* length of stay, *GOS* Glasgow Outcome Score, *LQ* lower quartile, *UQ* upper quartile

Table 3 delineates characteristics of the β-blocked (BB^(+)^) cases and their respective controls (BB^(−)^). There were no statistical differences in any variables included in the analysis. No discrepancies were noted with regard to patient characteristics, intracranial injury severity, occurrence of specific types of injury, or required neurosurgical interventions (Table 3). Unfavorable long-term functional outcomes (GOS ≤ 3) were detected in 50.7% of the total cohort (Table 2). Following propensity score matching, the mean NICU LOS in the BB^(+)^ group and in the BB^(−)^ control group was 8.5 ± 11.7 vs. 10.8 ± 9.1 days (*p* = 0.09). The mean hospital LOS was shorter in the BB^(+)^ cohort (18.0 days vs. 26.8 days, *p* < 0.01) (Table 4). There was no difference in unfavorable GOS at discharge between the two cohorts (88.2% for BB^(+)^ vs. 89.5% for BB^(−)^, *p* = 1.00). At follow-up, however, the BB^(+)^ group experienced significantly fewer cases of poor long-term functional outcome (GOS ≤ 3) than their BB^(−)^ counterparts, with 42.1 vs. 59.2%, respectively (*p* = 0.03) (Table 4). The risk of poor long-term functional outcome was more than doubled in non-β-blocked patients (OR 2.44, 95% CI 1.01–6.03, *p* = 0.03). Table 5 depicts the breakdown of GOS scores between BB^(+)^ and BB^(−)^ patients at discharge and at follow-up.

**Table 3:** Demographics and clinical information prior to and following propensity score matching

	Before matching			After matching		
	BB^(−)^	BB^(+)^	*p*	BB^(−)^	BB^(+)^	*p*
	(*n* = 286)	(*n* = 76)		(*n* = 76)	(*n* = 76)	
Patient characteristics						
Male gender	75.2% (215)	75.0% (57)	1.0	78.9% (60)	75% (57)	0.61
Age in years			<0.001			0.68
Mean (SD)	46.0 (18.6)	57.2 (16.3)		57.9 (15.8)	57.2 (14)	
Median (LQ, UQ)	47 (29, 62)	62 (50, 69)		62 (49, 69)	62 (50, 69)	
Age ≥ 55 years	39.5% (113)	71.1% (54)	<0.001	69.7% (53)	71.1% (54)	1.00
CCI			<0.001			0.70
Mean (SD)	1.9 (2.2)	3.5 (2.4)		3.4 (2.7)	3.5 (2.1)	
Median (LQ, UQ)	1 (0, 3)	3 (2, 5)		3 (1, 5)	3.0 (2, 5)	
Specific intracranial injury						
Diffuse axonal injury	11.5% (33)	5.3% (4)	0.12	5.3% (4)	5.3% (4)	1.00
Focal injury	16.4% (47)	13.2% (10)	0.60	10.5% (8)	13.2% (10)	0.73
Epidural hemorrhage	18.2% (52)	11.8% (9)	0.23	11.8% (9)	11.8% (9)	1.00
Subdural hemorrhage	37.1% (106)	63.2% (48)	<0.001	67.1% (51)	63.2% (48)	0.58
Subarachnoid hemorrhage	9.1% (26)	5.3% (4)	0.34	5.3% (4)	5.3% (4)	1.00
Injury severity						
Hypotension (<90 mmHg)	4.5% (13)	3.9% (3)	1.0	2.6% (2)	3.9% (3)	1.00
GCS ≤ 8	62.9% (180)	44.7% (34)	0.01	52.6% (40)	44.7% (34)	0.18
Head AIS ≥ 4	86.0% (246)	84.2% (64)	0.71	85.5% (65)	84.2% (64)	1.00
Non-head NISS ≤ 9	71.7% (205)	92.1% (70)	0.001	86.8% (66)	92.1% (70)	0.30
Rotterdam, median (LQ, UQ)	3 (3, 4)	4 (3, 4)		4 (3, 4)	4 (3, 4)	
Neurosurgical interventions						
ICP monitoring	69.2% (198)	64.5% (49)	0.49	63.2% (48)	64.5% (49)	1.00
Operation	44.1% (126)	60.5% (46)	0.01	48.7% (37)	60.5% (46)	0.18

*BB* β-blockade, *GCS* Glasgow Coma Scale, *CCI* Charlson’s Comorbidity Index, *AIS* Abbreviated Injury Scale, *NISS* New Injury Severity Score, *ICP* Intracranial Pressure, *LQ* lower quartile, *UQ* upper quartile

**Table 4:** Outcomes prior to and following propensity score matching

Outcomes	Before matching			After matching		
	BB^(−)^	BB^(+)^	*p*	BB^(−)^	BB^(+)^	*p*
	(*n* = 286)	(*n* = 76)		(*n* = 76)	(*n* = 76)	
NICU LOS			0.03			0.09
Mean (SD) days	11.1 (9.5)	8.5 (8.3)		10.8 (9.1)	8.5 (11.7)	
Median (LQ, UQ) days	8.4 (3.0, 17.0)	5.0 (3.0, 12.0)		8.0 (3.0, 16.0)	5.0 (3.0, 12.0)	
Hospital LOS						
Mean (SD), days	24.2 (18.7)	18.0 (12.6)	<0.01	26.8 (21.7)	18.0 (24.9)	**<0.01**
Median (LQ, UQ), days	19 (10, 32)	15 (8, 24)		21 (9, 34)	15 (8, 24)	
In-hospital mortality	6.3% (18)	11.8% (9)	0.14	7.9% (6)	11.8% (9)	0.58
12-month Mortality	14.0% (40)	22.4% (17)	0.08	26.3% (20)	22.4% (17)	0.70
GOS ≤ 3 at discharge	88.5% (253)	88.2% (67)	1.00	89.5% (68)	88.2% (67)	1.00
GOS ≤ 3 at follow-up	42.7% (122)	42.1% (32)	1.00	59.2% (45)	42.1% (32)	**0.03**

*BB* β-blockade,* GCS* Glasgow Coma Scale,* CCI* Charlson’s Comorbidity Index,* AIS* Abbreviated Injury Scale,* NISS* New Injury Severity Score,* ICP* Intracranial Pressure, *LQ* lower quartile, *UQ* upper quartile

**Table 5:** Functional outcomes at discharge and follow-up in the matched cohorts

Functional outcome	GOS at discharge			GOS at follow-up		
	BB^(-)^	BB^(+)^	*p*	BB^(-)^	BB^(+)^	*p*
	*n* (%)	*n* (%)		*n* (%)	*n* (%)	
GOS ≤ 3	68 (89.5)	67 (88.2)	1.00	45 (59.2)	32 (42.1)	0.03
GOS 1	6 (7.9)	9 (11.8)		14 (18.4)	13 (17.1)	
GOS 2	4 (5.3)	0 (0)		1 (1.3)	0 (0)	
GOS 3	58 (76.3)	58 (76.3)		30 (39.5)	19 (25.0)	
GOS 4	8 (10.5)	9 (11.8)		17 (22.4)	28 (36.8)	
GOS 5	0	0		14 (18.4)	16 (21.1)	

*GOS* Glasgow Outcome Score

## Discussion

The current study observed that severe TBI cases subjected to early and continuous β-blocker therapy had a significantly better long-term functional outcome compared to controls without BB exposure. To the authors’ best knowledge, there are no previous investigations evaluating the effect of β-blockade on functional outcome following severe TBI.

During the last decade, β-blockade has been suggested as a protective agent following injury supported by the results from numerous studies [11–17]. The hypothesized mechanism of β-blockade in TBI, not yet clearly elucidated, is the ability to block the hyperactivity of sympathetic adrenoceptors. The ‘sympathetic storm’ following traumatic brain injury has been linked to worse neurologic outcome with the increasing catecholamine levels of serum or urine been associated with increased in-hospital mortality [22]. Murine models of TBI have demonstrated that the administration propranolol significantly reduces trauma-induced cerebral edema and results in improved neurologic recovery [23]. It has been observed that rising catecholamine levels in the early phases of head injury correlate with worsening GCS and GOS, suggesting that sympathetic hyperactivity is detrimental to survival and functional recovery [24]. Nevertheless, clinical studies have so far focused on in-hospital survival but not on long-term functional outcome.

We noted that a total of 24.3% of patients died within the 12-month study period and 9.9% of the deaths occurred during the in-hospital phase of care after excluding patients with non-survivable injuries (death within 48 h of admission). Our findings also indicate that the cohort of initial survivors experienced a poor early functional outcome with 88.8% (*n* = 135/152) assigned a GOS of ≤ 3 at discharge and 50.7% (n = 77/152) at follow-up. Likewise, there was no significant difference between the BB^(+)^ and BB^(−)^ groups on analysis of GOS at discharge. However, the β-blocker exposed cohort did have a significantly shortened mean hospital LOS (18.0 ± 24.9 vs. 26.8 ± 21.7 days, *p* < 0.01).

Interestingly, a significant long-term benefit on functional outcome was noted with a two-fold reduction of poor outcome measured by GOS (OR 2.44, 95% CI 1.01–6.03, *p* = 0.03) up to 1 year after injury. Consequently, early initiation of β-blocker therapy and continuous exposure throughout the hospital stay appears to have a therapeutic role in long-term neurological recovery. We speculate that an extended recovery span is required beyond the hospital LOS for improved outcomes after severe TBI, typically through multi-interventional neuro-rehabilitation efforts.

Previous studies have demonstrated that pre-admission and in-hospital β-blocker exposure is associated with improved in-hospital survival following TBI [14, 25]. While there is an obvious overlapping relationship between mortality and neurologic recovery, there was no difference between BB^(+)^ and BB^(−)^ groups in terms of in-hospital mortality (11.8 vs. 7.9%, *p* = 0.58) or overall mortality (up to 12 months) (26.3 vs. 22.4%, *p* = 0.70). We speculate that this is a consequence of selection bias introduced by the exclusion of unavoidable deaths due to non-survivable injury (deaths ≤ 48 h of admission), the smaller sample size, and the reduction in confounders through propensity score matching. Patients were matched according to variables, such as age, injury severity (AIS, NISS, Rotterdam CT score and GCS) and comorbidity (Charlson’s Comorbidity Index), which are individually known to affect survival perspectives following traumatic injury. As a result, the potential for mortality differences between the sub-groups is reduced.

To the best of our knowledge, this is a pioneering investigation observing long-term benefits of β-blocker administration following severe TBI. Nevertheless, there are several limitations to the study. First, while the median time for long-term GOS assessment was close to 12 months, some patients had the assessment later as well as earlier. Through our experience, these discrepancies result in very small variations in the data and thus represent only a minor limitation. Second, while GOS is the gold standard for functional assessment, it might be too crude to assess complaints related to post-traumatic stress disorder or quality-of-life assessment, which we believe should be the subject of future studies [26, 27]. Third, secondary cerebral injuries or other complications during the admission period or after discharge, which invariably have an impact on long-term functional outcome, could not separately be accounted for in the current study. Finally, the precise cause of death could not be determined.

## Conclusion

Early and continuous exposure to β-blocker medication in patients with severe TBI appears to improve functional outcomes. Further prospective randomized trials and investigation into the underlying mechanisms of this effect are warranted.

## Abbreviations

TBI: Traumatic brain injury
GCS: Glasgow Coma Scale
ISS: Injury Severity Score
IRB: Institutional review board
AIS: Abbreviated Injury Scale
NICU: Neurosurgical Intensive Care Unit
LOS: Length of stay
SBP: Systolic blood pressure
CT: Computer tomography
MRI: Magnetic resonance imaging
NISS: New Injury Severity Score
GOS: Glasgow Outcome Score
ICU: Intensive Care Unit
SD: Standard deviation
OR: Odds ratio
CI: Confidence interval
BB: Beta-blocker
